# Laparoscopic cholecystectomy induce tension pneumocephalus in a patient with ventriculoperitoneal shunt: A case report and literature review

**DOI:** 10.1097/MD.0000000000035967

**Published:** 2023-11-10

**Authors:** Tan-Si Chu, Tan-Huy Chu, Tri-Dung Huynh, Hoang-Vu Mai, Van-Dinh Phan, Bao-Ngoc Dang, Quoc-Dat Tran, Xuan-Sang Le

**Affiliations:** a Department of Neurosurgery, Tam Anh Hospital, Ho Chi Minh City, Vietnam; b Department of Hematology, Pham Ngoc Thach University of Medicine, Ho Chi Minh City, Vietnam.

**Keywords:** abdominal pressurization, laparoscopic-induced pneumocephalus, ventriculoperitoneal shunt

## Abstract

**Introduction and patient concerns:**

We report on a 45-year-old woman who has a ventriculoperitoneal shunt (VPS), experienced drowsy mental status, with hypesthesia and hemiplegia on the left side. Ten days ago she underwent laparoscopic cholecystectomy (LC). Computed tomography revealed tension pneumocephalus, with severe compression on the right side of the brain.

**Interventions and diagnosis:**

She underwent 2 surgeries, the first surgery was to place a subdural drainage catheter, however, the pneumocephalus relapsed after withdrawing the catheter, and the later surgery was to replace the new VPS.

**Outcomes:**

After replacing the VPS, the patient recovers completely after 10 weeks of follow-up.

**Conclusion:**

To our knowledge, this is the first report of LC-induced tension pneumocephalus in a patient with VPS. The purpose of this study is to share our experience, with the hypothesized mechanism being the retrograde air through the VPS valve because of high abdominal pressurization. We recommend noting the existence of the VPS when the LC or any abdominal laparoscopy is performed. The VPS should be clamped during any laparoscopic procedure until complete depressurization. Furthermore, all patients with VPS who have neurological deterioration after abdominal laparoscopy should be treated as having the diagnosis of a tension pneumocephalus. These patients need emergency surgery to replace VPS and set the valve for high-pressure, which can result in a quick and complete recovery.

## 1. Introduction

Hydrocephalus occurs when there is an obstruction in the normal outflow or a decreased absorption of the cerebrospinal fluid (CSF), and affected around 400,000 children worldwide annually.^[1]^ Most affected patients are untreated due to limited access to the medical system, which leaves an irreversible effect on neurodevelopment and can result in death. In patients that have access to medical care, ventriculoperitoneal shunt (VPS) currently is the main option to treat hydrocephalus, which is a cerebral shunt that drains excess CSF from ventricular to peritoneal.^[1]^

Laparoscopic cholecystectomy (LC) is a minimally invasive surgical procedure for the removal of a diseased gallbladder. This technique is one of the most common abdominal surgical procedures and essentially has replaced the open technique for routine cholecystectomies since the 1990s.^[2]^ The previous studies reported that LC is considered safe and major complications occur in <3% of patients, with the most significant common complication being an injury to the bile duct.^[2,3]^ Here, we reported an extremely rare case of LC-induced tension pneumocephalus in a patient with a VPS, which was successfully treated by surgery that replace the VP shunt. Additionally, we also provide the hypothesized reasons for pneumocephalus and recommendations for management and treatment.

## 2. Case presentation

**Figure 1.**
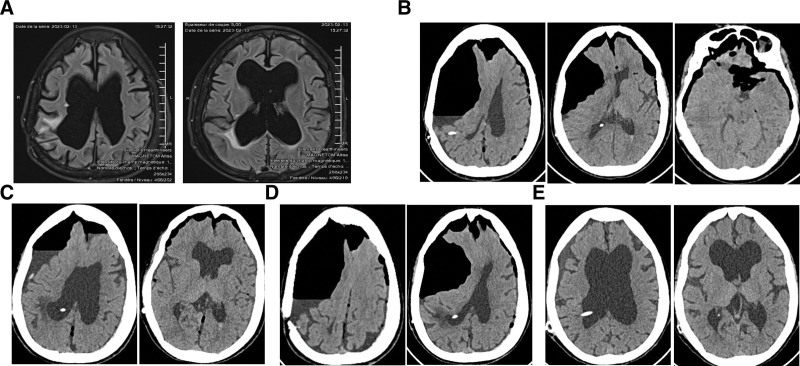
(A) Before the LC, brain MRI revealed reveal no abnormalities; (B) 10 days after the LC, CT revealed tension pneumocephalus, with severe compression on the right side of the brain; (C) 24 hours after the first surgery to place a subdural drainage catheter, the CT reveal reduced compression of the brain; (D) 96 hours after the first surgery and 24 hours after withdrawing the catheter, the CT scan revealed relapse tension pneumocephalus with severe compression of the right brain; (E) 72 hours after the second surgery to replace the VPS, the CT reveal no sign of pneumocephalus. All the images are in axial view. CT = computed tomography, LC = laparoscopic cholecystectomy, MRI = magnetic resonance imaging, VPS = ventriculoperitoneal shunt.

We report on a 45-year-old woman who experienced headache, numbness and weakness on the left side of her body. Ten days ago, she underwent LC, the LC operation was a success, with abdominal pressure of 12 mm Hg. Before the LC, brain magnetic resonance imaging revealed reveal no abnormalities (Fig. 1A). Three days ago, she initially experienced a headache. The headache kept getting worse, along with increasing numbness and weakness on the left side of the body. She was admitted to Tam Anh Hospital, a facility that can diagnose and treat patients with neurological disorders using cutting-edge methods in Vietnam.^[4]^ She had a medical history of mild mental retardation, 20 years ago she experienced hydrocephalus and was treated with VPS; 2 years ago, she experienced hydrocephalus again because of obstruction of the VPS, and the new VPS was replaced.

On the physical examination, the blood pressure, pulse, temperature, and respiration rate were 120/70 mm Hg, 100/min, 37°C, and 20/min, respectively, the patient’s condition overall is poor. Neurological examination revealed a Glasgow Coma Scale score of 14 points (Eye 4, Verbal 4, Motor 6), drowsy mental status, hypesthesia and hemiplegia on the left side. In detail, the left hand and left foot muscle strength grading was grade 1 and 0, respectively. The Hoffman sign and the Babinski sign were both negative. Computed tomography revealed tension pneumocephalus, with severe compression on the right side of the brain (Fig. 1B). The patient underwent emergency surgery to eliminate the air, decompress the brain, and place a subdural drainage catheter. After the surgery, the patient was treated with ceftriaxone 1 g × 2 times/day (intravenous; IV), metronidazole 500 mg × 2 times/day (IV) for 7 days, and depakine 500 mg × 2 times/day (oral). At 24 hours after the first operation, the patient’s condition improved with a GCS of 15, and the CT reveal reduce compression of the brain (Fig. 1C). At 72 hours, the patient’s condition continue to improve with both the left hand and left foot muscle strength grading was 2, and the subdural drainage catheter was withdrawn. However, at 96 hours, the patient feel severe headaches with both the left hand and left foot muscle strength grading was 1, the CT scan revealed relapse tension pneumocephalus with severe compression on the right brain (Fig. 1D). The patient underwent a second emergency surgery to replace the VPS (Strata II, Medtronic, USA). We also set the valve for high-pressure to accumulate CSF in the ventricles, increase the pressure inside and push the brain to the outside, eliminating the space that can cause air accumulation. The CT scan after the second surgery showed a decrease of pneumocephalus. At 168 hours (7 days) after the first operation, the patient’s condition improve with both the left hand and left foot muscle strength grading was grade 4, the scan showed no sign of pneumocephalus (Fig. 1E), and the patient is discharged. After 10 weeks of follow-up since hospital admission, the patient recovers completely.

## 3. Discussion

Pneumocephalus is the presence of intracranial air. Despite the fact that pneumocephalus is regularly observed on routine imaging following craniotomy in neurosurgical practice, there are few publications regarding pneumocephalus that are available, the majority of which are case reports with little epidemiological information.^[5]^ The general causes of pneumocephalus in 75% of patients are neurotrauma, in the other 25% are infections, tumors, cranial and spinal procedures.^[5,6]^ Pneumocephalus is mostly asymptomatic, however, it can cause headache, nausea, seizures and even focal neurological symptoms.^[5]^ It is important to differentiate between a pneumocephalus and a tension pneumocephalus; given the benign nature of pneumocephalus the treatment is usually conservative, however, a tension pneumocephalus is considered as a neurological emergency.^[5,7]^

To the best of our knowledge, this is the first report of LC-induced tension pneumocephalus in a patient with VPS. The VPS is a versatile procedure to treat patients with hydrocephalus, which consist of a proximal catheter in the ventricular, a one-way valve to prevent backflow and can change pressure, and a distal catheter placed in the peritoneal to absorb CSF. The peritoneal cavity is often chosen, since it is relatively easy to access, can absorb large volumes of CSF, and can place extra tubing to allow for the growth of patients.^[1]^ The VPS is not a contraindication of LC. Around 30% of VPS placed in infants fail in the first year, 40% fail in the second year, and up to 90% fail by 10 years. VPS can fail for various reasons, however, the most common causes are obstruction and infection rather than valve dysfunction.^[1]^ In a prior report, a patient with VPS had laparoscopic-induced pneumocephalus, the authors hypothesized the changes in the valve of the VPS placed 20 years ago, allowed the backflow through the valve.^[6]^ Nevertheless, in our case, the VPS was replaced 2 years prior, making it less likely the main reason. Thus, we hypothesized the main mechanism behind this phenomenon is the high abdominal pressurization by the LC forced retrograde air through the VPS valve and breaking the valve. According to an in vitro study, retrograde reflux will not occur during laparoscopic abdominal surgery when insufflation pressures range from 3 to 25 mm Hg,^[8]^ however in this case, the abdominal pressure was 12 mm Hg and still force retrograde air through the VPS valve and break it; thus, we recommend clamp the VPS during any laparoscopic procedures until complete depressurization. In this case, the first surgery was to place a subdural drainage catheter, still, the pneumocephalus relapsed after withdrawing the catheter which is proof that the VPS valve was broken. Realizing this problem, we performed a second emergency surgery to replace the new VPS and set the valve for high-pressure to accumulate CSF in the ventricles, increase the pressure inside and push the brain to the outside, reducing the space that can cause air accumulation. The hypothesis of retrograde air breaking the valve in VPS, and replacing early after suspected the cause results in the patient’s quick and complete recovery. We recommend noting the existence of the VP shunt when performing any abdominal laparoscopy. The VPS should be clamped during any laparoscopic procedures until complete depressurization. All patients with VPS who have neurological deterioration after abdominal laparoscopy should be treated as having the diagnosis of a tension pneumocephalus, and early replacement of the VPS is recommended. The limitation of this study is that we can only hypothesize the mechanism in a single case, due to the rarity of patients with VPS undergoing abdominal laparoscopy. If we have a chance, we will perform the recommended steps in this study to test our hypothesis in future similar cases.

In conclusion, in this study we presented an immensely rare case of tension pneumocephalus as a complication of LC in a patient with VPS, the main mechanism is the retrograde air through the valve in the VPS because of high abdominal pressurization. We recommend noting the existence of the VP shunt when performing the LC or any abdominal laparoscopy, especially in those patients with an old shunt. The VPS should be clamped during any laparoscopic procedures until complete depressurization. Furthermore, all patients with VPS who have neurological deterioration after abdominal laparoscopy should be treated as having the diagnosis of a tension pneumocephalus. These patients need emergency surgery to replace VPS and set the valve for high-pressure, which can result in a quick and complete recovery.

## Acknowledgments

We would like to say our special thanks to Quynh Nguyen Xuan Thuy, Department of Infectious Diseases, Children’s Hospital No. 2, Ho Chi Minh City, Vietnam; and Tan-Son Chu, Department of Medicine, Nguyen Tat Thanh University, Ho Chi Minh City, Vietnam; for their contribution to English correction, and literature review.

## Author contributions

**Conceptualization:** Tan-Si Chu, Tan-Huy Chu.

**Data curation:** Tan-Huy Chu.

**Formal analysis:** Tan-Huy Chu.

**Funding acquisition:** Tan-Si Chu.

**Investigation:** Tan-Si Chu, Tan-Huy Chu, Tri-Dung Huynh, Hoang-Vu Mai, Van-Dinh Phan, Bao-Ngoc Dang, Quoc-Dat Tran, Xuan-Sang Le.

**Methodology:** Tan-Si Chu, Tri-Dung Huynh, Hoang-Vu Mai, Van-Dinh Phan, Bao-Ngoc Dang, Quoc-Dat Tran, Xuan-Sang Le.

**Project administration:** Tan-Si Chu, Tan-Huy Chu.

**Supervision:** Tan-Si Chu, Tan-Huy Chu.

**Visualization:** Tan-Si Chu.

**Writing—review & editing:** Tan-Huy Chu.

**Writing—original draft:** Tan-Huy Chu.
